# Altered hierarchical organization between empathy and gambling networks in disordered gamblers

**DOI:** 10.3389/fpsyt.2023.1083465

**Published:** 2023-02-09

**Authors:** Hui Zhou, Yuwen He, Zhen Yuan, Yuan Zhou, Jingwen Yin, Robin Chark, Davis Ka Chio Fong, Lawrence Hoc Nang Fong, Anise M. S. Wu

**Affiliations:** ^1^Department of Psychology, Faculty of Social Sciences, University of Macau, Macao, Macao SAR, China; ^2^Centre for Cognitive and Brain Sciences, Institute of Collaborative Innovation, University of Macau, Macao, Macao SAR, China; ^3^Bioimaging Core, Faculty of Health Sciences, University of Macau, Macao, Macao SAR, China; ^4^CAS Key Laboratory of Behavioral Science, Institute of Psychology, Chinese Academy of Sciences, Beijing, China; ^5^Department of Integrated Resort and Tourism Management, Faculty of Business Administration, University of Macau, Macao, Macao SAR, China

**Keywords:** empathy, gambling disorder, resting-state fMRI, effective connectivity, brain networks, dynamic causal modeling

## Abstract

**Background:**

Despite the demonstrated association between empathy and gambling at the behavioral level, limited neuroimaging research on empathy and gambling disorder (GD) has been conducted. Whether and how the brain network of empathy and that of gambling interact in disordered gamblers has not been investigated. This study aimed to address this research gap by examining the hierarchical organizational patterns, in which the differences of causal interactions of these networks between disordered gamblers and healthy controls were revealed.

**Methods:**

Resting-state functional magnetic resonance imaging (fMRI) data of 32 disordered gamblers and 56 healthy controls were included in the formal analysis. Dynamic causal modeling was used to examine the effective connectivity within and between empathy and gambling networks among all participants.

**Results:**

All participants showed significant effective connectivity within and between empathy and gambling networks. However, compared with healthy controls, disordered gamblers displayed more excitatory effective connectivity within the gambling network, the tendency to display more excitatory effective connectivity from the empathy network to the gambling network, and reduced inhibitory effective connectivity from the gambling network to the empathy network.

**Conclusion:**

The exploratory study was the first to examine the effective connectivity within and between empathy and gambling networks among disordered gamblers and healthy controls. These results provided insights into the causal relationship between empathy and gambling from the neuroscientific perspective and further confirmed that disordered gamblers show altered effective connectivity within and between these two brain networks, which may be considered to be a potential neural index for GD identification. In addition, the altered interactions between empathy and gambling networks may also indicate the potential targets for the neuro-stimulation intervention approach (e.g., transcranial magnetic stimulation).

## 1. Introduction

Gambling disorder (GD) has been defined as a persistent and recurrent problematic gambling pattern, which leads to clinical impairment or distress (1). According to the dual-processing model of decision-making (2, 3), individuals with GD may have deficits in both the impulsive system and the reflective system (4, 5), and imbalance between these two neurocognitive systems may lead them gradually to become addicted to gambling (6, 7). This study aimed to address the knowledge gap regarding the association between empathy and GD from the neurocognitive perspective (8).

Empathy, which has been defined as the ability to share emotions similar to others’ experiencing (i.e., emotional empathy) and take others’ perspectives (i.e., cognitive empathy) (9, 10), might play a protective role in the development of GD. In decision-making, emotional empathy allows individuals to feel what others are feeling, leading them to make better decisions for others from the altruistic perspective, whereas cognitive empathy helps them predict others’ mental states and make better decisions for themselves from a self-interest perspective (11). Thus, empathy might help individuals balance their impulsive and reflective systems by holding them back from making impulsive decisions and helping them to make more rational decisions (12, 13). In addition, individuals’ deficits in empathy would also increase their vulnerability to social isolation and emotional distress, which may drive them to adopt addictive behaviors, including gambling, in order to regulate the associated negative emotions (14–16). On the other hand, disordered gamblers tend to be less aware of their thoughts and emotions (17); indeed, ignorance about how one is feeling is associated with weaker empathic ability (18). A recent scoping review (8) pointed out that there was better empirical evidence for the association of empathy with gambling behaviors than with GD because only one empirical study had tested empathy in disordered gamblers. The study found that disordered gamblers showed the reduced level of empathy in the self-report measure and the worse perspective-taking task performance compared to their healthy counterparts (19). Considering the scarce knowledge, further research with other methodologies, including neuroimaging, should be conducted for investigating the relationship between empathy and GD from various perspectives. Indeed, the reduced level of empathy have been consistently observed in substance-related disorders (20–25) but whether and how empathy and behavioral addiction are linked is understudied. The knowledge about such links, if any, can benefit not only the understanding addiction in general but also interventions for GD. Thus, more research attention on empathy and GD, including the analysis of the connectivity of their brain networks from a neural perspective, is warranted.

There are plenty of neuroimaging studies revealing the neural substrates of empathy and gambling individually. The empathy network includes two neural circuits, namely, the emotional contagion system and perspective-taking system (26). When individuals observe others’ emotions and behaviors, the emotional contagion system automatically activates neural responses, which will be reactivated by similar experiences. For example, functional magnetic resonance imaging (fMRI) studies have shown consistently that when participants observe others’ facial expressions (e.g., pleasure, disgust, and pain), their inferior frontal gyrus (IFG) and insula are automatically activated (27–29). The emotional contagion system seems to be involved in basic processing, whereas cognitive perspective-taking involves a higher-order function. Brain regions, such as the medial prefrontal cortex (mPFC), the superior temporal sulcus, and the temporal poles, have been found to be involved when individuals engage in tasks related to understanding and inferring others’ thoughts, goals, and behaviors (30, 31). These studies have provided evidence that IFG, insula, mPFC, superior temporal sulcus, and temporal poles are the core brain regions of empathy.

For addiction-related behaviors (e.g., gambling in our case), another model of dual-processing neural systems is involved (7). First is the impulsive system, which automatically facilitates the processing of gambling-related cues and increases gamblers’ cravings. Several fMRI studies have found that, compared with healthy controls, disordered gamblers show altered activations in the orbitofrontal cortex (OFC), anterior cingulate cortex (ACC), and ventral striatum when they view gambling-related pictures and videos (32–35) and when they are waiting to see the outcomes of their gambling decisions (36). Another crucial neural system is the reflective system, which involves reflection and efforts to control oneself from a long-term perspective. For example, altered responses in OFC and ACC were found in disordered gamblers in inhibition tasks compared to healthy controls (37, 38). The striatum and OFC are important neural regions in reward prediction and value encoding (39, 40), and ACC is a core brain region in saliency processing and cognitive control (41). Despite the versatile functions of these brain regions, altered activations have been frequently found in both the left and right striatum, OFC, and ACC in those with GD while performing different tasks.

Despite the intimate correlation between empathy and gambling behaviors at the behavioral level, no published research appears to have examined whether and how empathy-related and gambling-related brain networks interact with each other. Determining how these networks influence each other may help us uncover the neural underpinnings of the relationship between empathic ability and gambling behaviors at the behavioral level. To achieve this, we need to define the empathy network and the gambling network for further analysis. As we summarized above, specific brain regions have been frequently identified during empathy tasks or among disordered gamblers. It was expected that the automatic meta-analysis could be conducted to identify empathy and gambling brain networks.

Whereas some studies have reported interactions among the frequently reported brain regions involved in empathy and/or gambling-related tasks (42–47), no study to date has examined the interactions between empathy and gambling regions at the network level. Whether there is a hierarchical organization among these two brain networks is also unknown. Dynamic causal modeling (DCM) can help determine how the activity of a particular brain region exerts on another brain region (48, 49) and thus capture the directed interactions between brain regions. Specifically, it can help determine whether a brain region has excitatory effects or inhibitory effects on another brain region. For example, DCM was used to show more inhibitory effects of the right posterior parietal cortex on the right insula among patients with bipolar disorder than the healthy controls and this difference was taken as evidence for the stronger inhibitory connection between these two brain regions as a potential source of bipolar disorder (50). Thus, DCM is a proper tool to explore how the brain nodes of empathy and gambling networks interact within and between networks. In addition, by utilizing a Bayesian contrast strategy (51), taking account of both the mean and uncertainty of regions of interest (ROIs)-based level causal interactions to calculate the network level causal interactions, we were able to summarize the hierarchical organization between these two functional networks.

In summary, this study aimed to explore the directed interactions between functional networks of empathy and gambling in a sample including both disordered gamblers and healthy controls who took MRI scans. We defined empathy and gambling networks by the automatic meta-analysis provided by Neurosynth. With a case-control study design, we hypothesized that there was significant effective connectivity within empathy and gambling networks as well as between networks of empathy and gambling in the common-group level (Hypothesis 1). In addition, compared with the healthy controls, the disordered gamblers were hypothesized to be significantly different in effective connectivity among brain nodes of within and between networks of empathy and gambling (Hypothesis 2). DCM was used to explore the directed interactions among brain regions of these two functional networks identified. Furthermore, Bayesian contrasts were adopted to reveal the hierarchical organization of empathy and gambling networks.

## 2. Materials and methods

### 2.1. Participants and procedures

The online and offline recruitment advertisements for eligible participants (who are Chinese adults aged ≥ 18 years) were posted in the corresponding author’s university, centers of the local non-governmental organizations for social services, and general communities. Moreover, the referral of participants by Sheng Kung Hui Macau Social Services Coordination Office, a non-governmental organization which particularly provided counseling services for gamblers and families, was also adopted in the recruitment procedure. Individuals interested in this project would contact with the trained research assistant, who then arranged each participant’s schedule for participation in this study (i.e., clinical interview, self-reported questionnaire survey, and MRI scan).

Before taking the MRI scan, all the participants were interviewed by a psychiatrist, who had more than 5 years of experience on diagnostic interview of mental disorders at the psychiatric department of a public hospital in China. In this study, exclusion criteria included any self-reported physical disease, psychosis resulting from general medical conditions, mental illnesses (e.g., major depression, bipolar disorder, manic episode, and anxiety disorder), other severe medical conditions (especially taking dopaminergic medications and substance abuse), and/or family history of psychosis. According to the psychiatrist’s diagnostic assessment for participants’ past-year gambling experience, 32 participants were classified into the disordered gambler group and 57 participants were assigned to the healthy control group. All these participants were right-handed, but the data of one of them was removed from further analyses due to severe head motion during the MRI scan.

All participants were also asked to asked to fill out a questionnaire with the items regarding their demographics (e.g., sex and age) and GD symptoms (i.e., nine items listed in the fifth edition of the Diagnostic and Statistical Manual of Mental Disorders) (1, 52, 53). With a yes-no response scale, they reported whether they had experienced any of the nine symptoms (e.g., “Has made repeated unsuccessful efforts to control, cut back, or stop gambling.”) during the past year. Finally, our valid sample for data analyses consisted of 32 disordered gamblers (28 males/4 females) and 56 healthy controls (46 males/10 females). The average age of the gamblers was 38.27 (±12.68) years, whereas the average age of the healthy controls was 24.50 (±4.72) years (Table 1); moreover, the effects of sex and age were controlled for in all analyses. In addition, the average score of GD symptoms of the disordered gamblers was 5.63 (±2.61), while that of the healthy controls was 0.98 (±1.69), as shown in Table 1. The study acquired ethics approval from the Panel on Research Ethics (Sub-panel on Biomedical Science and Engineering) of the corresponding author’s university (reference number: BSERE20-APP014-ICI-01). Every participant gave their written consent before taking part in the study.

**TABLE 1:** Demographic information of participants.

	Disordered gambler	Healthy control	Statistics
Number	*N* = 32	*N* = 56	–
Age	38.28 ± 12.68	24.50 ± 4.72	*t* = 7.30, *p* < 0.001
Gender (male/female)	28/4	46/10	*K* = 0.44, *p* = 0.51
Gambling disorder symptom	5.63 ± 2.61	0.98 ± 1.69	*t* = 10.12, *p* < 0.001

### 2.2. MRI acquisition

MRI scans were obtained by a 3T Siemens Magnetom Prisma scanner (Siemens Healthineers, Erlangen, Germany). Every participant undertook a structural MRI scan for approximately 4 min and a resting-state functional MRI scan for 10 min. The parameters for the structural scan included that field of view (FOV) = 256 mm; slice number = 176; time of repetition (TR) = 2,300 ms; time of echo (TE) 2.26 ms; slice thickness = 1 mm. During the resting-state, the participants were instructed to fixate on a cross on a screen with a black background, focusing on no particular thing. The parameters for resting-state fMRI included that FOV = 192 mm; slice number = 65; slice thickness = 2 mm; TR = 1 s; TE = 30 ms; phase encoding direction = anterior-posterior (AP); flip angle = 60.

### 2.3. MRI data preprocessing

The preprocessing procedures of fMRI data were conducted by Data Processing and Analysis for (resting-state) Brain Imaging (DPABI) (54), a package based on MATLAB packages statistical parametric mapping (SPM) (55). Five volumes were removed from the original 600 volumes of resting-state MRI data for each participant to remove the effects of unstable factors at the beginning of the scan session. A total of 595 volumes of data were fed to perform realignment for head motion. The confounding effects, including constant, linear, and quadratic trends (56), 24 head motion parameters (57), the effects originated from white matter and cerebral fluid, as well as the regressors indicating bad head motion time points, were removed for further procedures. Before smoothing the data with a 4 mm Gaussian kernel, the data were normalized to a standard MNI space, taking advantage of each participant’s structural scan.

### 2.4. Regions of interest selection

The automatic meta-analysis provided by Neurosynth^1^ was used to generate the ROIs of empathy and gambling networks. The meta-analysis method used by Neurosynth is different from activation likelihood estimation (ALE) (58), or multi-level kernel density meta-analysis (MKDA) (59, 60). Neurosynth offers to conduct a two-way ANOVA testing for the presence of a non-zero association between a given term and voxel activation, while both ALE and MKDA are required to construct a null model by permutation methods. Compared with ALE and MKDA, Neurosynth is more time-saving and offers online automatic analysis. In addition, Neurosynth covers a broad range of journals, which could reduce the selection bias for included papers compared to manual searching and manual selection. In this study, empathy and gambling networks were defined to include the activated voxels, which have significant correlations with the corresponding terms empathy and gambling, respectively. Due to the various number of papers included in the meta-analysis of the terms, empathy and gambling, different sizes of clusters were generated for empathy and gambling networks. We cut the cluster size threshold for the empathy network at 150 voxels, whereas the threshold for significant clusters of the gambling network was above 50 voxels.

### 2.5. Extraction of volume of interest

The time series of each volume of interest (VOI) were extracted for each participant. Discrete cosine sets (ranging from 0.0078 to 0.1 Hz) were extracted as regressors to conduct a general linear model (GLM) to obtain voxels showing low-frequency fluctuations (uncorrected *P* < 0.05). In addition, all the voxels of an ROI within a gray matter mask were created as a group mask, in which the identified low-frequency voxels were included as VOI. At last, the principal eigenvariate for each VOI was extracted for further DCM analysis.

### 2.6. Dynamic causal modeling

Spectral DCM is a robust approach to exploring the causal connectivity among brain nodes in resting-state fMRI data (49). All the spectral DCM analyses were conducted with the DCM version 12.5 package of SPM version 7771. A fully connected model was created for each participant, after which all the parameters were estimated by a Bayesian model inversion method based on the observed cross-spectral density (49). The participants with explained model variance below 60% were removed for further analysis. After estimating the parameters of effective connectivity within each subject, a parametric empirical Bayes (PEB) framework (61, 62) was used to collate and model the parameters at the second level. During the same time, regressors, including diagnostic groups, age, sex, and head motion, were part of the PEB. The Bayesian evidence for all models were compared in order to select the best parameter set to explain the between-subject effects. To summarize the group-common and difference effects, Bayesian model averages were estimated to represent the group-average connection strengths (posterior probability, *Pp* > 0.90, indicating strong evidence for the connection strength).

To summarize the effective connections on a network level, we adopted the method used in previous studies (50, 51) to construct Bayesian contrasts to calculate the network-level average connection strengths instead of taking the arithmetic mean of the connection parameters. By utilizing a Bayesian contrast approach to obtain average connection strengths, the uncertainty (variance) of parameters could be considered. The posterior probability for Bayesian contrasts is used to indicate the kind of probability the average connection strength differs from 0. A posterior probability greater than 90% indicates strong evidence to suggest the existence of a network-level connection. This approach was taken to verify whether the effective connectivity within and between networks existed.

## 3. Results

### 3.1. ROIs selection for empathy and gambling networks

**FIGURE 1:**
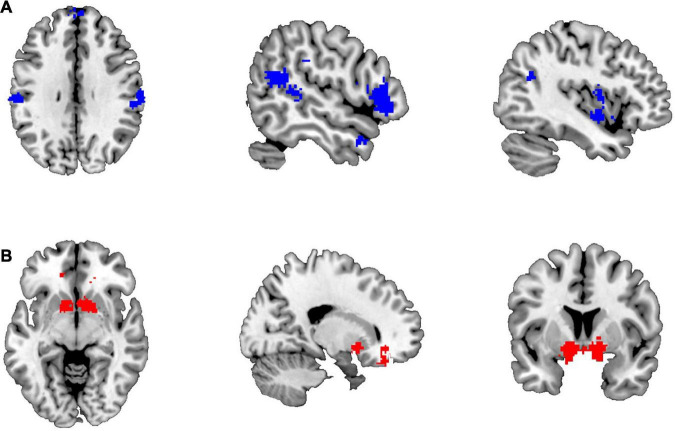
The brain regions identified for empathy and gambling networks. **(A)** The brain regions of the empathy network; **(B)** the brain regions of the gambling network.

There were 187 studies included for meta-analysis for the empathy network on Neurosynth, whereas only 85 studies were included for the gambling network. Eight brain blue regions (Figure 1A), including left postcentral gyrus (L-postcen), left insula (L-ins), right supramarginal gyrus (R-SupraMarg), left superior temporal gyrus (L-STG), left mPFC (L-mPFC), right IFG (R-IFG), right temporal pole (R-tempole), and right superior temporal gyrus (R-STG), were identified for the empathy network, whereas four red brain regions (Figure 1B), including bilateral striatum (i.e., L-striatum and R-striatum), left ACC (L-ACC), and right OFC (R-OFC) were identified for the gambling network.

### 3.2. Functional connectivity of empathy and gambling networks

**FIGURE 2:**
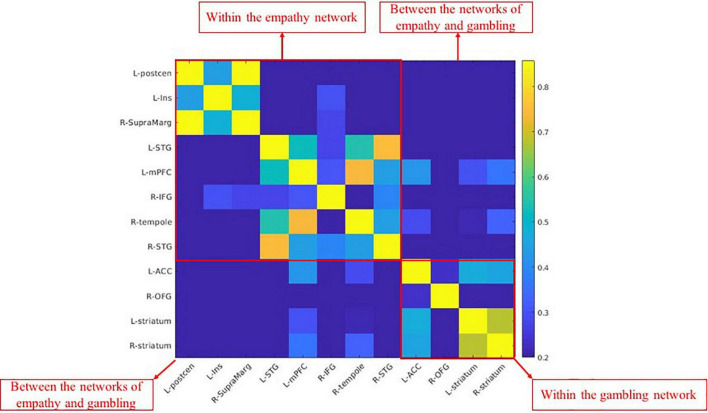
The functional connectivity matrix of empathy and gambling networks.

As displayed in Figure 2, the within-network functional connections were higher than the between-network functional connections. This finding suggests that the brain networks revealed by online automatic meta-analysis are potentially similar to the brain networks partitioned by some clustering methods, such as the principal component analysis, showing stronger within-network connectivity.

### 3.3. Effective connectivity

**FIGURE 3:**
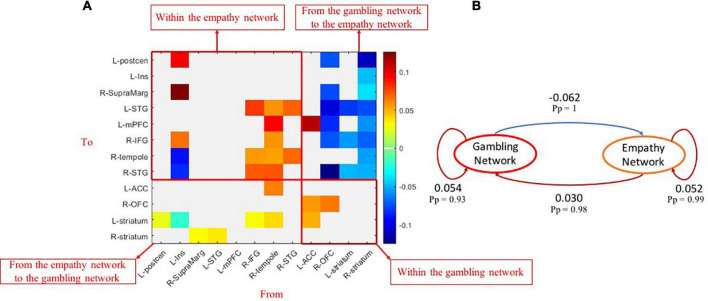
Group-common effects of effective connectivity among empathy and gambling networks. **(A)** The causal connections among brain nodes of empathy and gambling networks (*Pp* > 0.90); **(B)** the network-level common causal connections among empathy and gambling networks. *Pp*, posterior probability.

As shown in Figure 3A, the group-common within-empathy and within-gambling networks’ effective connectivity showed strong evidence (*Pp* > 0.90) of being nearly excitatory. The brain nodes of the empathy network had excitatory effects on the brain nodes of the gambling network, while the brain nodes of the gambling network had inhibitory effects on the brain nodes of the empathy network across all participants. These patterns were demonstrated on a network level (Figure 3B). Specifically, the group-common average effective connectivity of the empathy network was 0.052 (*Pp* = 0.99), while the group-common average effective connectivity of the gambling network was 0.054 (*Pp* = 0.93). Effective connectivity of both within-empathy and within-gambling networks was excitatory. It was also revealed that the group-common effective connectivity from the empathy network to the gambling network was excitatory 0.030 (*Pp* = 0.98), whereas the group-common effective connectivity from the gambling network to the empathy network was inhibitory (effective connectivity = −0.062, *Pp* = 1).

**FIGURE 4:**
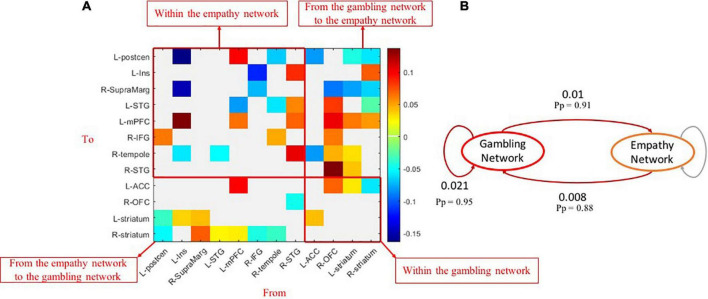
Group-different effects of effective connectivity among empathy and gambling networks. **(A)** The causal connections revealing group differences (*Pp* > 0.90) among brain nodes of empathy and gambling networks; **(B)** the network-level group differences of causal connections among empathy and gambling networks. *Pp*, posterior probability.

Compared with controls, those with GD showed increased or decreased excitatory effective connectivity among brain nodes of within-empathy and within-gambling networks (Figure 4A). Similar mixed patterns were also displayed in group different effective connectivity between empathy and gambling network brain nodes. At the network level, within the gambling network, excitatory effective connectivity increased in disordered gamblers (Figure 4B, *Pp* = 0.95), whereas, in comparison to healthy controls, no group differences in effective connectivity were found at the network level within the empathy network. One should note that the gamblers had marginally significant increased excitatory effective connectivity from the empathy network to the gambling network (*Pp* = 0.88) but reduced inhibitory effective connectivity from the gambling network to the empathy network (*Pp* = 0.91), which might contribute to higher activations within the gambling network.

It should be noted that gender did have significant effects on the causal interactions within and between empathy and gambling networks (*Pp* = 0.91–0.98) at the network level of analysis. Therefore, the effects of age, gender and head motion, as covariates, were controlled for the group-common and group-difference effects in all the aforementioned results.

## 4. Discussion

The present study appeared to be the first to investigate the effective connectivity of empathy and gambling brain networks in disordered gamblers and healthy controls during rest. This exploratory study revealed the common hierarchical organization of empathy and gambling brain networks in both groups. In addition, it demonstrated that compared to healthy controls, the hierarchical organization of empathy and gambling brain networks in disordered gamblers was altered, which may be a potential characteristic index for GD.

Our neuroimaging data enriched the existing literature on the relationship between empathy and gambling, which has been mainly tested at the behavioral level. Consistent with Hypothesis 1, our results further revealed that the underlying brain networks of empathy and gambling have a specific hierarchical organization. We demonstrated that the excitatory connections within the empathy network and the gambling network were shared by participants with GD and healthy controls. The results of group-common effects of connectivity within empathy and gambling networks were generally consistent with previous functional connectivity studies in healthy participants. Specifically, the reported functional connectivity pattern of the insula (63) showed that the insula was positively correlated with IFG, STG, and postcentral gyrus (sensorimotor areas), and the reported functional connectivity pattern of the temporal pole (64) also revealed the positive correlations between the temporal pole and STG, mPFC, and IFG. In addition, there were positive correlations among ACC, OFC, and parts of the striatum (42, 65, 66). The current findings also indicated that the empathy network tends to excite the gambling network, whereas the gambling network inhibits the activations of the empathy network. These results echoed findings not only in the recent scoping review (8), pointing out the association between empathy and gambling, but also in previous empirical studies showing the positive correlations between temporal pole and ACC, OFC (64) and the negative correlations between the striatum and IFG, postcentral gyrus, supramarginal gyrus, and STG (65). In addition, a recent study by Zhang et al. (67) also found that brain areas involved in self-awareness and introspection (e.g., mPFC) had excitatory effects on the subcortical reward network (e.g., the striatum). Thus, our study expanded the knowledge regarding the bidirectional relationship between empathy and gambling brain networks, which may provide some insights for elucidating how empathy and GD influence each other on the behavioral level.

This study also found that participants with GD, compared with healthy controls, showed altered effective connectivity within the gambling network and between empathy and gambling networks. These results support Hypothesis 2 and are congruent with previous studies that showed that functional connectivity between reward-related networks (e.g., ACC and striatum) was stronger in the participants with GD in contrast to the healthy controls (68, 69). In addition, one effective connectivity study also reported that individuals with higher scores on disinhibition and impulsivity, which are common deficits in disordered gamblers, had lower inhibitory activity in the self-connection of OFC (70). Despite impaired empathy in disordered gamblers (19), one should note that the interactive organization within the empathy network of our disordered gambler group could not be significantly differentiated from that of our healthy control group. Further research must further examine the replicability of these findings across ages and cultural groups.

In addition to disordered gamblers’ increased effective connectivity within the gambling network, we also found that the participants with GD had a marginally significant increase in excitatory effects from the empathy network to the gambling network but reduced inhibitory effects from the gambling network to the empathy network, compared to the healthy controls. One should note that the increase excitatory effects and reduced inhibitory effects between empathy and gambling networks do not necessarily indicate higher or lower behavioral level related to empathy and GD. We only can speculate that all of these subtle but significant changes of causal interactions between empathy and gambling networks, together with the increased excitatory connections among brain nodes within the gambling network of disordered gamblers, contribute to the excessive activations of the gambling network, provided that other conditions remain unchanged. To our best knowledge, no previous study has explored the hierarchical organization of empathy and gambling brain networks in participants with GD. Despite a significant portion of studies showing a decrease in activation in the brain regions of the gambling network in those with GD (71, 72), a number of other studies have revealed an increase in activation in disordered gamblers, compared with healthy controls, during task performance. These studies have shown similar findings: positive correlations between disordered gamblers’ subjective craving for gambling or impulsivity scores and the activations of caudate or ACC (33, 73); a positive relationship between activations of ACC and the striatum and indifference to delayed reward in GD (74, 75); greater activation of ACC in relation to response inhibition performance in disordered gamblers (38); and increased activation of the OFC, right caudate when participants were engaged in high-risk gambling tasks (76). All of these results indicated that higher activations of OFC, ACC, striatum, and caudate predicted more severe levels of GD. Our findings supported the premise that higher activations within the gambling networks may stem from the altered hierarchical organization between empathy and gambling networks. Future studies are also recommended to make clear how the altered hierarchical organization between networks of empathy and gambling among disordered gamblers is associated with their disordered behavioral patterns.

Because empathy helps individuals make more rational decisions (12, 13), the empathy system may function to maintain a balance between gamblers’ impulsive and reflective systems. Moreover, the impaired interaction patterns between the empathy and impulsive systems, as well as reflective systems, is likely to disrupt this balance and exacerbate one’s vulnerability to GD. These two systems play important roles in GD from the bottom up and top down, respectively (77, 78), and more effective connectivity studies are recommended to incorporate these two pathways to understand the mechanisms of GD and design the interventions for GD. In our current study, the core brain regions involved in reward processing and cognitive control that comprise the gambling network are important components of the hypothesized impulsive and reflective systems at the neural level. The revealed hierarchical organization of empathy and gambling brain networks in disordered gamblers, compared to healthy controls, showed that the empathy network could readily excite the gambling network (marginally significant), while there were weakened inhibitory effects from the gambling network to the empathy network. These disruptive interactions between empathy and gambling networks, together with the tendency for increased excitatory connections within the gambling network in the GD group, might, at least in part, contribute to this group’s higher activations in the gambling network, which underlie the deficits in disordered gamblers. The disruptive pattern of effective connectivity between empathy and gambling networks may also be the potential neural indicator for early screening for GD. Furthermore, examining whether the disruptive interactions between empathy and gambling networks can be ameliorated by targeting those brain regions with altered interaction patterns during transcranial magnetic stimulation or other neuro-stimulation therapies may provide a promising direction for designing an effective integrated program for GD intervention.

Several limitations of this study should be noted. First, the smaller sample size of the GD group compared to the control group might reduce statistical power. However, compared with past neuroimaging studies in GD (see the reviews) (71, 72), our sample size of disordered gamblers was relatively large, particularly considering the difficulty in reaching them due to their reluctance to seek help for GD (79). Second, there were more male participants than female participants in our study because of the higher GD prevalence among males than females (80). Therefore, future studies are warranted that incorporate a larger sample of disordered gamblers, probably with a cross-regional and cross-cultural design, with a similar male-to-female ratio in order to test whether reproducible results are obtained. Last but not least, trait impulsivity, which has been identified as one of the major pathways to GD (14, 81), was not measured in the present study. Considering that the core brain regions of the gambling network involved important brain areas of impulsivity, future studies are recommended to incorporate trait impulsivity into the analysis (e.g., the interactions between empathy and gambling networks are correlated with trait impulsivity) to further elucidate how the brain regions and their connectivity patterns observed are associated with GD.

In conclusion, our exploratory study appeared to be the first to examine the common hierarchical organization of empathy and gambling brain networks in disordered gamblers and healthy controls, and it also compared the causal relationship within and between empathy and gambling networks between these two groups. Results revealed the hierarchical organization of empathy and gambling networks, which provided useful insights into the bidirectional relationships between empathy and gambling and/or its disorder. In addition, the disruptive pattern of this hierarchical organization between empathy and gambling networks has the potential to be used as a neural marker for screening disordered gamblers. Also, the altered brain regions are the promising targets for neuro-stimulation interventions if the findings are reliably replicated in the larger-scale research in the future.

## Data availability statement

The raw data supporting the conclusions of this article will be made available by the authors, without undue reservation.

## Ethics statement

The studies involving human participants were reviewed and approved by the Panel on Research Ethics (Sub-panel on Biomedical Science and Engineering) of the University of Macau (reference number: BSERE20-APP014-ICI-01). The patients/participants provided their written informed consent to participate in this study.

## Author contributions

HZ: conceptualization, writing—original draft, and writing—reviewing and editing. YH: methodology, formal analysis, and writing—original draft. ZY: funding acquisition and writing—reviewing and editing. YZ: methodology and writing—reviewing and editing. JY and DKCF: methodology. RC and LHNF: writing—reviewing and editing. AMSW: conceptualization, funding acquisition, supervision, and writing—reviewing and editing. All authors contributed to the article and approved the submitted version.

## Footnotes

1
https://neurosynth.org/
